# Crohn's Disease in a Saudi Outpatient Population: Is it Still Rare?

**DOI:** 10.4103/1319-3767.45357

**Published:** 2009-04

**Authors:** Mohammad A. Al-Mofarreh, Ibrahim A. Al Mofleh, Ibrahim N. Al-Teimi, Abdulrahman M. Al-Jebreen

**Affiliations:** Dr. Al- Mofarreh Polyclinic, King Saud University, Riyadh, Saudi Arabia; 1 College of Medicine, King Saud University, Riyadh, Saudi Arabia; 2 King Saud Medical Complex, Ministry of Health, Riyadh, Saudi Arabia

**Keywords:** Crohn's disease, increasing trend, Saudi Arabia

## Abstract

**Background/Aim:**

To determine the epidemiology of Crohn's disease (CD) in an outpatient clinic and compare it with data previously reported from different centers in the Kingdom of Saudi Arabia and outside.

**Materials and Methods:**

The medical records of all patients with CD seen in the clinic in the period from January 1993 through December 2007 were reviewed. The demographic, clinical data and methods of diagnosis were retrieved.

**Results:**

Over a period of 15 years, we saw 133 Saudi patients with CD. They were predominantly young, with a median age of 26.2 years and male preponderance (2.3:1). The final diagnosis was established within 1 week of presentation in 47% of the patients. The leading symptoms were abdominal pain (88%), diarrhea (70%), bloating (61%), rectal bleeding (50%), weight loss (33%), constipation (24%) and perianal disease (23%). The diagnosis was established by endoscopy and histopathology. Ileocecal involvement was encountered in 40% of the patients.

**Conclusion:**

From the current study, it is obviously possible to diagnose a large proportion of patients with CD in a gastroenterology outpatient clinic. The data revealed a strikingly increased incidence of CD in a mainly young Saudi population in the past few years.

The pathological and clinical entity of Crohn's disease (CD) has been known to clinicians since the description of the disease by Crohn ***et al*** in 1932.[1] It is a chronic, recurrent, transmural, granulomatous inflammation of the bowel and may affect any site of the gastrointestinal tract (GIT).[2] Despite tremendous work concerning autoimmune, genetic, environmental and infectious factors, the etiology remains obscure.[3–6] Epidemiological studies have shown that CD tends to increase worldwide, particularly in industrialized countries with geographical variations.[7–11] Few reports have been published in the last three decades from the Kingdom of Saudi Arabia (KSA) and Kuwait indicating that it used to be a rare disease in this part of the world.[12–15] However, recent reports from various centers in the KSA have shown an increasing incidence.[16–19]

The aim of this study is to report for the first time CD from a gastroenterology-oriented polyclinic in Riyadh, KSA, and contribute in collecting epidemiological data of this inflammatory bowel disease entity.

## MATERIALS AND METHODS

This is a retrospective study of all patients diagnosed as CD and seen in the period from January 1993 to December 2007 at the Al-Mofarreh Polyclinic, a gastroenterology-oriented private clinic receiving patients from all over the KSA. All medical records of patients with CD were thoroughly reviewed by senior consultant gastroenterologists from different institutions. Demographic, clinical, laboratory, endoscopic, histopathologic and imaging data were analyzed. The history was taken in all patients with special reference to dietary habits. Clinical examination and laboratory investigations were analyzed. Complete blood count, erythrocyte sedimentation rates (ESR), C-reactive protein (CRP), bilirubin, alanine aminotransferase, alkaline phosphatase, creatinine, stool and urine examinations were performed in all patients, while iron, total iron binding capacity (TIBC), electrolytes, anti-***Saccharomyces cerevisae*** antibody (ASCA) and perinuclear antineutrophilcytoplasmic antibody (p-ANCA) were performed selectively. Stool culture was performed when indicated to rule out infectious causes and a purified protein derivative (PPD) test was performed when tuberculosis was considered in the differential diagnosis. All 133 patients had lower gastrointestinal endoscopy (LGIE) and 39 of them, who simultaneously had upper gastrointestinal symptoms, were also subjected to upper gastrointestinal endoscopy (UGIE) with documentation in all cases. Multiple biopsies were obtained from affected and apparently normal sites. Enteroclysis, which should have been routinely performed for a proper documentation of the location, extent and severity of the disease, was declined by nine patients due to financial reasons. For the same reasons, abdominal ultrasonography, computed tomography (CT)-scan and magnetic resonance imaging examination were performed selectively. The final diagnosis was achieved by endoscopy with positive histopathology. Nine patients, although evident by imaging, had a nonspecific or negative histopathology and two patients treated initially as CD and finally diagnosed as intestinal tuberculosis were not included in the study.

## RESULTS

Over a period of 15 years, a total of 39,720 patients attended the clinic. Thirty-seven thousand seven hundred and thirty-four patients had gastrointestinal symptoms and 3,201 (8%) underwent LGIE. One hundred thirty-three (0.36%) Saudi patients had CD. All were diagnosed endoscopically with positive histopathology. Only three patients (2%) were seen in the first 8 years of the study period (1993–2000) whereas 130 (98%) were seen in the second 7 years (2001–2007) and 64% of all patients presented in the last 2 years alone.

**Figure 1:**
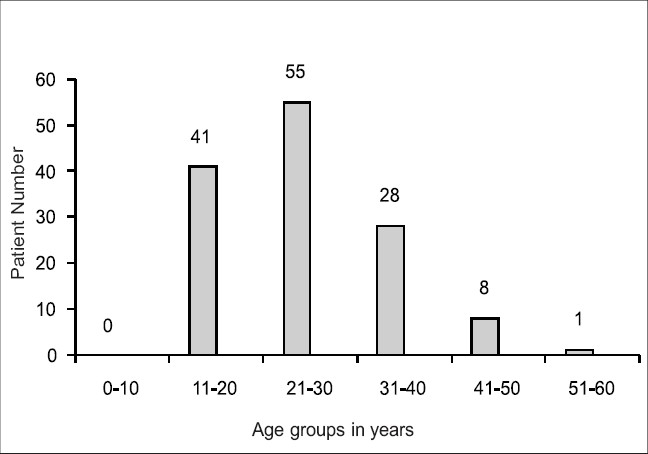
Age distribution (n: 133)

One hundred and three patients were primarily diagnosed and treated as CD in our clinic and seven elsewhere. Another 23 patients, diagnosed and treated elsewhere as ulcerative colitis (UC), actually had CD. The median age was 26.2 years and male gender was more frequently affected (2.3:1) [Table 1]. Age distribution is shown in Figure 1. The leading symptoms were abdominal pain (88%), diarrhea (70%), bloating (61%), rectal bleeding (50%), weight loss (33%), constipation (24%) and perianal disease (23%). The duration of symptoms before presentation obtained in 126 patients exceeded 12 months in 72% of them. However, the lapse between presentation and final diagnosis was less than 3 months in 66% [Table 2]. Moreover, the final diagnosis was established within 1 week of presentation in 47%. Twelve patients (9%) had extraintestinal manifestations in the form of arthralgia (seven patients), erythema nodosum, raised transaminases, sclerosing cholangitis, ascites and amenorrhea (one each).

**Table 1:** Demographic characteristics of CD patients

Demographic data	Number of patients (%)
Total number	133 (100)
Males	95 (71)
Females	42 (32)
Male : female ratio	2.3:1
Age range (years)	11–54
Median age (years)	26.2
Nationality (Saudi)	133 (100)

CD: Crohn's disease

**Table 2:** Duration of symptoms of patients aware of the duration (*n* = 126)*

Duration (months)	< 3	3–6	7–12	>12
Symptoms to presentation	14 (11)	15 (12)	6 (5)	91 (72)
Presentation to suspected diagnosis	102 (81)	3 (2.5)	3 (2.5)	18 (14)
Presentation to final diagnosis	83 (66)	12 (9)	5 (4)	26 (21)

* Seven patients could not remember when their symptoms started. Figures in parentheses are in percentages.

The main abnormal laboratory findings were decreased hemoglobin (37%), raised ESR and CRP level (36%) and thrombocytosis (>400,000) in 22% of the patients. The most common abnormal findings in stool examination were white blood cells (32%), mucus (32%) and positive blood (25%).

**Figure 2:**
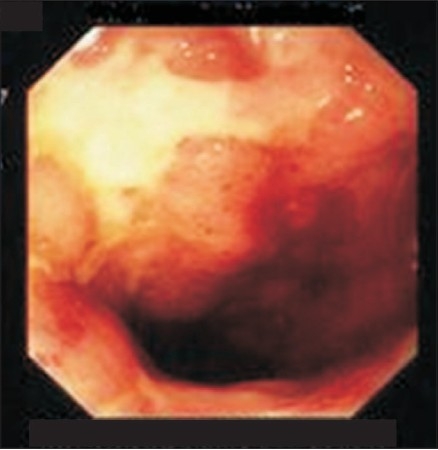
Deep map-like terminal ileum ulcer and thickened nodular mucosa

**Figure 3 (a and b):**
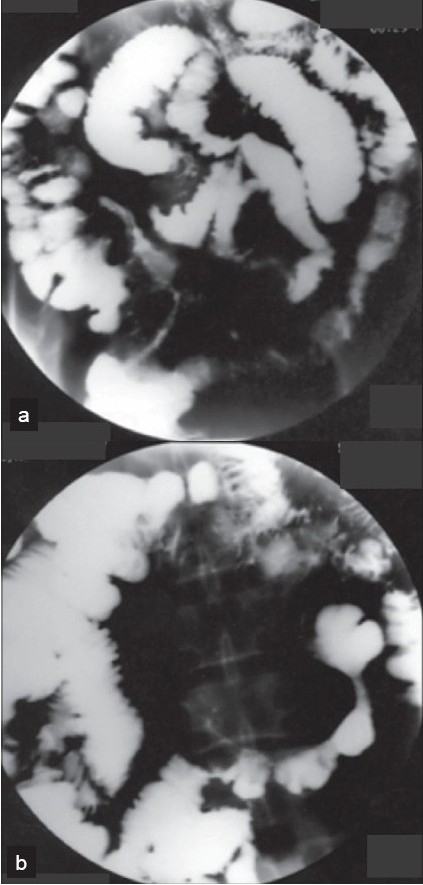
Small bowel radiology with skip lesions, string sign and entroenteric fistula

The frequently encountered colonoscopic findings were erythema, apthal ulcers and erosions in 71, 55 and 48%, respectively [Table 3], [Figure 2]. For various reasons including severe colitis, stenosis and procedure intolerance, the cecum was not always amenable for visualization. Positive colonic histopathology along with radiological features of CD were used to identify those with ileocolonic involvement. Important histopathological criteria constituted mixed inflammatory cells (71%), transmural inflammation (71%), dilated lymphatics (11%) and granulomas (9%). Enteroclysis was performed in our clinic in most of the CD patients and also in UC patients with inappropriate response to treatment. The most common findings of enteroclysis in 124 CD patients were thickening, ulcerations, cobble stoning/nodularity, narrowing and bowel separation in 59, 40, 34, 30 and 29%, respectively. Other radiological features are shown in Table 4 and Figure 3a and Figure 3b. Both the ileum and the large intestine were affected in 40% of the patients whereas the colon alone was involved in 17% and the ileum alone in 29%. The upper GIT was involved in 14%. According to the Vienna classification of CD, “any disease location proximal to the terminal ileum with or without additional involvement of the terminal ileum or colon has been classified as upper GIT-involvement [L4].”[20]

**Table 3:** Colonoscopic findings in CD (*n* = 133)

Colonoscopic findings	Number of patients (%)
Erythema	95 (71)
Apthal ulcers	73 (55)
Erosions	64 (48)
Cobble stoning/nodularity	40 (30)
Thickening	21 (16)
Friability	17 (13)
Perianal fistula (active)	7 (5)
Normal finding	5 (4)

CD: Crohn's disease

**Table 4:** Radiological findings of enteroclysis in CD (*n* = 124)

Radiological findings	Number of patients (%)
Wall thickening	73 (59)
Ulcerations	49 (40)
Cobble stoning/nodularity	42 (34)
Narrowing	37 (30)
Bowel separation	36 (29)
Fistulas	24 (19)
Pseudosacculation	12 (10)
Marked segmentation	8 (6)
Nonspecific/normal	19 (15)

CD: Crohn's disease

The final diagnosis was established by endoscopy and positive histopathology. Radiological features of CD were encountered in 85% and 73% of the patients examined by enteroclysis and CT-scan, respectively [Table 5].

**Table 5:** Methods of diagnosis of CD (*n* = 133)

Methods	Number of procedures	Positive (%)	Non specific (%)	Negative (%)
LGIE	133	131 (98)	1 (1)*	1 (1)*
LGI–histopathology	133	129 (97)	4 (3)**	0.0
Enteroclysis	124	105 (85)	5 (4)	14 (11)
UGIE	39	13 (33)	26 (67)	0.0
UGI–histopathology	27	11 (41)	16 (59)	0.0
CT-scan	22	16 (73)	6 (27)	0.0

* These patients had positive histopathology.

** These four patients had positive UGI–histopathology

## DISCUSSION

CD is a common disease in industrialized countries with an increasing incidence worldwide. Before 25 years, it was believed that CD does not exist in the KSA. The first two cases were reported in 1982 by Mokhtar and Khan.[12] Five years later an additional three cases were reported by Mohamed ***et al.***[14] Subsequently, more reports with an increasing number have appeared in the literature.[15,17–19,21,22] Of these, the largest series constituting 77 patients, all Saudi nationals except four, was published in 2004 by Al-Ghamdi ***et al.***[19] All of these studies originated from large medical centers.

In contrast, the present series is the first outpatient-based report from a gastroenterology-oriented polyclinic in Riyadh, **KSA**. One hundred and three patients were primarily diagnosed and treated in the clinic whereas the remaining 30 patients, who attended the clinic for follow-up, were initially diagnosed and treated elsewhere as CD (seven patients) and 23 patients were misdiagnosed as UC. Our study, constituting only Saudi patients, concurs with ***Al-Ghamdi et al.***'s[19] study including 92% Saudi nationals and both show a tremendous increase of CD in the KSA over the past10 years and obviously more over the last few years of the study period.

The increasing number of patients with CD over the last two to three decades is associated with drastic changes in environmental factors, education, occupation, income and industrialization as well as the increasing consumption of nicotine, oral contraceptives and nonsteroidal anti-inflammatory drugs, which have been reported as possible risk factors of CD.[5,23] An increasing adoption of the fast-food culture as was found in the majority of our predominantly young patients, while consuming an inadequate breakfast in the form of croissants, cakes, chocolates or sandwiches from the next take-away restaurant. Westernization of food habits may play a major role in the increasing incidence of the disease in the younger Saudi generation. This matter needs to be subjected to intensive nation-wide studies.

Beside environmental factors, genetic factors are also supposed to contribute to the pathogenesis of inflammatory bowel disease. First-degree relatives have an approximately 15 times higher risk than the general population.[24] CD and UC are related polygenetic diseases that share some susceptibility genes with environmental and hereditary factors. The influence in genetics is stronger in CD than in UC. The NOD-2 gene plays a role in overactivating NF-k1 in monocytes.[3,4] The CARD 15 gene as a complex gene-gene interaction increases the risk of CD.[25] It might be of interest to see our patients subjected to such investigations in future studies.

In our series, the disease affected mainly a younger age group of patients with a median age of 26.2 years. Population-based studies from Western countries have shown a relatively higher median age of 30 years.[26] This is in concordance with other studies from the **KSA**.[18,19] In pediatric patients, El Mouzan ***et al.*** have reported 19 cases of CD. The age of the majority (89%) ranged between 13 and 18 years.[22] The male preponderance rate in our study is comparable with some reports from the KSA and Japan[21,10] and is higher than other reports from the KSA, USA and Europe.[18,19,27,28]

In concordance with other studies, abdominal pain, diarrhea and weight loss were the leading presenting symptoms.[18,19] Hematochezia, usually not a common feature of CD, was encountered in 43% of our patients and was higher than reported previously from the KSA.[19] Similarly, perianal disease that was comparable to Western studies[27] was higher than reported in previous studies from the KSA and Kuwait.[13,18,19] Stomatitis, glossitis and apthal ulcers, not reported previously from the KSA, were the main presenting symptoms in 4% of our patients. There is a paucity of information about the duration of symptoms before the diagnosis. However, Mekhijian ***et al.*** reported in 1979 in the National Cooperative Crohn's Disease Study that half of all CD patients have symptoms that date 6 months or less,[29] whereas 75% of our patients were diagnosed within 6 months or less and 79% were first diagnosed in the clinic in < 1 year. Moreover, 47% were diagnosed in the first week, indicating the importance of a high index of physician and patient awareness.

Similar to other studies, anemia was the main laboratory finding, with a notable decrease in the serum iron level followed by ESR and CRP elevation, depending on the severity and the duration of the disease.[18,19] Some laboratory tests may prove useful in differentiating CD from UC. Dubinsky ***et al.*** have reported that CD-specific ASCA and UC-specific p-ANCA could enhance the diagnostic accuracy and minimize the need for invasive investigations, particularly in pediatric age group.[30]

Periappendicular involvement has been reported in 75% of the patients with CD of the terminal ileum with skip areas in case of colonic involvement.[31] In our series, the terminal ileum [L1] alone was affected in 29% compared with 30–40% in the other series, while colonic involvement alone [L2] occurred only in 17%, which is less than other reports worldwide. Forty percent of our patients had simultaneous involvement of the terminal ileum and the large bowel [L3], which is comparable with other studies.[20,32–34] Upper GIT [L4] involvement was encountered in 14%, with half of them having duodenal involvement (7% of our patients), which concurs with other reports. For instance, Poggioli ***et al.*** have observed duodenal involvement in 3.6% of their patients mimicking a peptic ulcer disease, which tends to improve on CD treatment, or in the form of duodenal stenosis, which more often requires surgery.[35] Oberhuber ***et al.*** have found granulomas in 15% of the patients and observed focally enhanced gastric inflammation as a characteristic finding.[36] Upper GI involvement was endoscopically evident in 33% of the patients who had upper GI endoscopies (10% of all CD patients), giving a positive histopathology in 41% of the biopsied patients. We believe that this figure could be higher if all CD patients were subjected to UGIE with biopsies from suspected lesions. It might also be noteworthy considering CD in the differential diagnosis of patients with ulcers in the oral cavity.

In conclusion, the current, along with previously reported data, show a strikingly increased incidence of CD in the KSA. It affects mainly a younger age group with a male preponderance and is comparable with the disease worldwide. Westernization of food habits particularly by the younger population may play a pivotal role in the increasing incidence of the disease and should be subjected to extensive nationwide studies. Upper GI involvement seems to be not as rare as expected; hence, it is suggested to perform UGIE with biopsies from different sites in patients with CD. Enteroclysis and CT are recommended in all CD patients to assess the localization, extent and intensity of the disease and also in patients with UC with inappropriate response to treatment. A high index of gastroenterologist and patient awareness as well as improvement in the gastroenterology training and diagnostic facilities will contribute to the early diagnosis and appropriate management of CD.
